# Mechanistic Interplay Between Multiple Myeloma and Severe SARS-CoV-2 Infection: Therapeutic Promise of Mesenchymal Stem Cell-Derived Extracellular Vesicles

**DOI:** 10.3390/biomedicines14071617

**Published:** 2026-07-17

**Authors:** Yan Leyfman, Niharika Ikkurthy, Taha Kassim Dohadwala, Helena Sanchez Coloma, Jenna Ghazal, Muskan Joshi, Viviana Cortiana, Diksha Sanjana Pasnoor, Gayathri P. Menon, Noam Levi, Maduri Balasubramanian, Chandler Park

**Affiliations:** 1 NewYork Presbyterian Hospital, Brooklyn, NY 11215, USA; yan.leyfman@nyp.org; 2 Cedars-Sinai Medical Center, Los Angeles, CA 90048, USA; niharika.ikkurthy@cshs.org; 3 Department of Medicine, David Tvildiani Medical University, 0159 Tbilisi, Georgia; 4 Harvard University, Cambridge, MA 02138, USA; helenacoloma@gmail.com; 5 Kalamazoo College, Kalamazoo, MI 49006, USA; ghazaljenna@gmail.com; 6 Faculty of Medicine, Tbilisi State Medical University, 0186 Tbilisi, Georgia; joshimuskan11@gmail.com (M.J.); gayathripramil20@gmail.com (G.P.M.); maduribalasubramanian@gmail.com (M.B.); 7 Department of Medical and Surgical Sciences (DIMEC), University of Bologna, 40126 Bologna, Italy; viviana.cortiana@studio.unibo.it; 8 Kamineni Academy of Medical Sciences and Research Centre, Hyderabad 500074, Telangana, India; dikshasreddy@gmail.com; 9 School of Physical Sciences, University of California, Irvine, CA 92697, USA; the.noam.levi@gmail.com; 10 Dudley Dunlap School of Biological Sciences, University of California, Irvine, CA 92697, USA; 11 Norton Cancer Institute, Louisville, KY 40202, USA; chandler.park@louisville.edu

**Keywords:** multiple myeloma, COVID-19, interleukin-6, extracellular vesicles, mesenchymal stem cells

## Abstract

Patients with multiple myeloma (MM) exhibit profound immune dysregulation, predisposing them to severe outcomes following SARS-CoV-2 infection. Current evidence highlights shared immunopathological mechanisms linking MM and COVID-19, with particular emphasis on the interleukin-6 (IL-6) axis as a shared amplifier of inflammation rather than the sole driver of disease. MM is characterized by a baseline pro-inflammatory milieu, in part mediated by IL-6, which is further amplified during SARS-CoV-2 infection, resulting in cytokine escalation, complement activation, coagulopathy, and multi-organ injury. This amplification operates within a broader, redundant network that also includes T-cell exhaustion, NK-cell dysfunction, checkpoint signaling, complement and endothelial injury, and treatment-induced immune defects. This overlap provides a mechanistic basis for the disproportionately high morbidity and mortality observed in this population. Even with advancements in vaccination, antiviral therapy, and clinical practice, patients with MM who exhibit impaired vaccine responses, active disease, or treatment-related immune dysfunction continue to experience considerable vulnerability to COVID-19. In addition, MM patients demonstrate suboptimal vaccine-induced immune responses, contributing to persistent vulnerability to severe and breakthrough infections. Modern MM therapies, including anti-CD38 antibodies, BCMA-directed agents, and bispecific T-cell redirecting antibodies, further reshape antiviral immunity by reducing NK cells, inducing plasma cell aplasia, causing hypogammaglobulinemia, and impairing T-cell function. Emerging treatment approaches targeting this shared pathway have been explored, with a focus on mesenchymal stem cell (MSC)-derived extracellular vesicles (EVs). EVs exhibit multimodal properties, including suppression of pro-inflammatory cytokines, restoration of immune homeostasis, inhibition of viral entry, and promotion of tissue repair and regeneration. Early clinical experience in severe COVID-19 populations suggests a favorable short-term safety profile; reported efficacy, however, derives from small, largely uncontrolled or early-phase studies, and the single randomized trial reporting a mortality benefit did so only in an exploratory post hoc subgroup, with its pre-specified primary endpoint not met. Overall, EVs constitute a biologically plausible but as yet unproven adjunctive strategy that warrants further investigation. Critically, no MM patient has ever been enrolled in an EV trial; current rationale for EV use in MM is therefore extrapolated entirely from non-MM populations, and no MM-specific data exist. Difficulties such as EV heterogeneity, manufacturing variability, uncertain pharmacokinetics, limited targeting efficiency, and potential prothrombotic effects must be resolved before their application in MM-specific clinical settings.

## 1. Introduction

Multiple myeloma (MM) is a clonal plasma cell malignancy characterized by the aberrant proliferation of terminally differentiated B cells within the bone marrow microenvironment [1]. A hallmark feature of MM is profound and multifaceted immunodeficiency affecting both innate and adaptive immunity, representing a primary driver of patient morbidity and mortality [2,3,4,5]. Infections constitute one of the leading causes of death in MM patients, with early mortality rates significantly influenced by infectious complications [6,7].

The immunologic dysfunction in MM spans both humoral and cellular arms: secondary antibody deficiency (typically hypogammaglobulinemia) coexists with impaired NK-cell cytotoxicity, T-cell compartment erosion, and defective dendritic-cell antigen presentation. Because these defects are treated functionally in Section 3.3, they are introduced here only to frame the central argument—that the breadth of immune compromise in MM, compounded by microenvironmental damage, demands a multimodal rather than single-target therapeutic approach [8,9].

This baseline immunodeficiency is frequently exacerbated by therapeutic interventions. Immunomodulatory agents, including thalidomide, lenalidomide, and pomalidomide, have been associated with an increased risk of severe infections [10,11]. Furthermore, advanced cellular immunotherapies such as chimeric antigen receptor T (CAR T)-cell therapy directed against B-cell maturation antigen (BCMA) induce profound plasma cell aplasia and sustained hypogammaglobulinemia, thereby exacerbating B-cell depletion and contributing to persistent impairment of humoral immune function [12,13].

The emergence of the SARS-CoV-2 pandemic markedly highlighted this clinical fragility. Early data demonstrated that MM patients experienced markedly worse outcomes compared to the general population, including disproportionately high rates of hospitalization, intensive care unit (ICU) admission, and mortality [14,15,16,17]. While vaccination remains the cornerstone of public health strategy, [14] studies have consistently demonstrated that patients with MM exhibit highly variable and often suboptimal humoral and cellular immune responses to SARS-CoV-2 vaccination [18,19,20,21].

Multiple clinical and treatment-related factors have been implicated in attenuated SARS-CoV-2 vaccine responses among patients with MM, including high-risk cytogenetics, ongoing disease activity, and receipt of intensive immunosuppression. In particular, exposure to targeted agents such as anti-CD38 monoclonal antibodies (daratumumab, isatuximab) and BCMA-directed therapies has been consistently associated with significantly reduced rates of both humoral and cellular immune responses following vaccination [18,22]. Notably, daratumumab-treated patients exhibit significantly reduced seroconversion rates following SARS-CoV-2 vaccination [18]. Consequently, even in the current endemic era, MM patients face substantial and persistent risk for breakthrough infections and severe SARS-CoV-2 disease [18,23].

The severity of COVID-19 in patients with MM is shaped in part by interleukin-6 (IL-6)-mediated mechanisms, though, as developed below, IL-6 is best understood as one amplifying node within a redundant inflammatory network. SARS-CoV-2 infection elicits a profound systemic inflammatory response characterized by elevated levels of IL-6, tumor necrosis factor-α (TNF-α), and interleukin-1β (IL-1β), which collectively contribute to the dysregulated cytokine release syndrome (“cytokine storm”) observed in severe cases and are associated with multi-organ dysfunction and adverse clinical outcomes [24,25,26]. IL-6 has been identified as a central biomarker of the hyperinflammatory response in COVID-19, with elevated serum levels consistently correlating with disease severity, respiratory failure, and adverse clinical outcomes [27,28].

Extensive evidence demonstrates that IL-6 acts through autocrine and paracrine signaling to promote proliferation, inhibit apoptosis, and facilitate malignant plasma cell expansion within the bone marrow microenvironment [29,30,31]. IL-6-induced signaling via JAK/STAT3 and related pathways also contributes to angiogenesis, osteoclast activation, and remodeling of the tumor niche, thereby supporting tumor progression and resistance to apoptosis [30]. Viral infections, which potently induce IL-6 as part of the antiviral response, may further amplify this baseline elevation, potentially exacerbating systemic inflammation and contributing to hyperinflammatory states, organ dysfunction, and worse clinical outcomes [32,33,34,35].

Given the evolution of COVID-19 outcomes with vaccination, antiviral therapy, and updated standards of care, the relevance of new interventions such as EVs should be evaluated within the current treatment setting rather than based solely on early-pandemic data.

In this comprehensive narrative review, we address the critical need for safe and effective therapeutic interventions for this vulnerable population. We first delineate the molecular and immunologic interplay between MM and severe COVID-19, organizing the shared pathophysiology into an explicit three-tier framework, (i) a predisposing baseline state, (ii) acute viral amplifiers, and (iii) therapy-imposed modifiers, so that the IL-6 axis is situated within, rather than above, the wider network. We then evaluate MSC-derived extracellular vesicles (EVs) as a cell-free therapeutic platform, contrasting their properties with parent MSCs and reviewing the clinical evidence for their use in severe SARS-CoV-2 infection in immunocompromised hosts, with particular emphasis on the MM population.

## 2. Materials and Methods

This article is an expert narrative review (rather than a systematic or scoping review) synthesizing the published literature on the interplay of MM and severe SARS-CoV-2 infection, with a focus on the therapeutic potential of mesenchymal stem cell-derived EVs. Because the aim was thematic integration across heterogeneous study designs rather than quantitative pooling or formal certainty grading, GRADE, AMSTAR, and PRISMA frameworks were not applied; evidence was instead synthesized thematically, prioritizing clinical relevance. The narrative format was chosen to allow for the inclusion of the maximal amount of data and to incorporate a broad range of study designs.

A search of PubMed/MEDLINE, Scopus, and Web of Science was conducted with no limits on year of publication, to allow for the maximal collection of data, given the rapidly evolving patterns of SARS-CoV-2 treatment and the development of EVs. Priority was given to studies published from 2019 onwards to reflect the changed MM treatment modalities after the onset of the SARS-CoV-2 pandemic. Conference abstracts, preprints, and non-English-language publications were excluded.

Search terms included the following key concepts and combinations: “multiple myeloma”, “SARS-CoV-2”, “COVID-19”, “extracellular vesicles”, “exosomes”, “mesenchymal stem cells”, “mesenchymal stromal cells”, “cytokine storm”, “immune dysregulation”, “immunotherapy”, and others. Both forward and backward snowballing were performed to identify relevant literature. Article selection and narrative synthesis were performed by the authors based on relevance to the central themes of the review. As article selection was based on author judgment rather than prespecified inclusion or exclusion criteria, the process may be subject to selection bias.

## 3. Pathophysiologic Synergy Between Multiple Myeloma and Severe SARS-Cov-2 Infection

**Figure 1:**
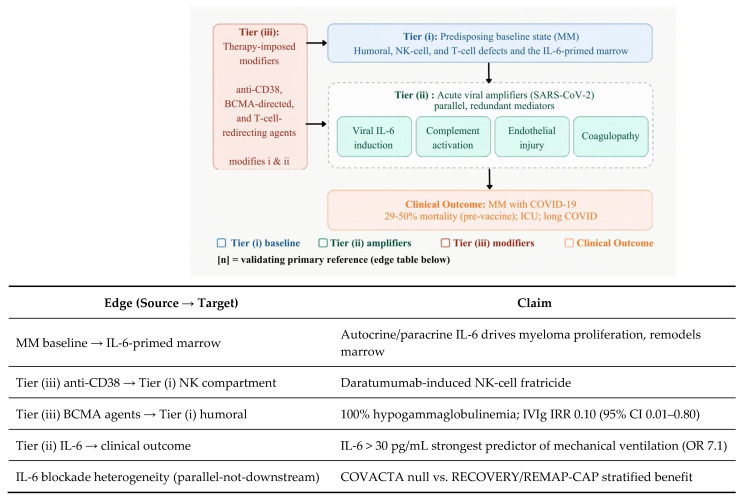
Three-tier mechanistic model of the interplay between multiple myeloma (MM) and severe SARS-CoV-2 infection. Tier (i), the predisposing baseline state (humoral, NK-cell, and T-cell defects; IL-6-primed marrow); Tier (ii), acute viral amplifiers (viral IL-6 induction shown as one of several parallel inputs alongside complement activation, endothelial injury, and coagulopathy); and Tier (iii), therapy-imposed modifiers (anti-CD38, BCMA-directed, and T-cell–redirecting agents).

Before surveying individual mechanisms, it is useful to state the organizing logic of this section explicitly because the central interpretive problem in this field is that mechanisms are typically catalogued rather than ranked. We propose that the synergy between MM and severe COVID-19 is best understood as a three-tier system. Tier (i), the predisposing baseline state, comprises the humoral, NK-cell, and T-cell defects and the IL-6-primed marrow that exist before any viral exposure. Tier (ii), the acute amplifiers, comprises the events that a SARS-CoV-2 infection superimposes on that substrate, viral IL-6 induction, complement activation, endothelial injury, and coagulopathy. Tier (iii), the therapy-imposed modifiers, comprises the distinct immune lesions created by anti-CD38, BCMA-directed, and T-cell-redirecting agents. The therapeutic argument advanced later in this review follows directly from this structure: MSC-derived EVs are proposed precisely because they act across tiers (i) and (ii) simultaneously, whereas no available agent, and no EV preparation, addresses tier (iii). The subsections below populate these tiers in turn (Figure 1).

### 3.1. Clinical Vulnerability of MM Patients to SARS-CoV-2

Individuals with MM who develop COVID-19 are positioned at an important intersection of increased vulnerability. The impaired immune function and the necessity for intensive therapeutic regimens present a challenge to COVID-19 treatment and increase the risk for greater severity of infection, more frequent hospitalizations, and higher mortality compared to the general population [16,36]. Within a large multicenter European cohort, the EPICOVIDEHA registry, data from 2020 demonstrated a significantly higher rate of overall and attributable mortality (31% and 22%) from COVID-19 among patients with hematological malignancies overall, figures that derive from the broader all-hematological-malignancy 2020 cohort rather than from MM patients specifically, compared to the general population’s COVID-19 mortality (0.1–9.4%) [37]. Similar outcomes were seen in a 2020 New York City multicenter cohort study of MM patients with COVID-19, which found a mortality rate of 29% [14]. While vaccination practices and advances in COVID-19 treatment regimens have reduced mortality and the risk of severe COVID-19 in recent years, individuals with hematological malignancies, and those with MM in particular, remain at higher risk compared to the general population [36,37,38].

Despite the overall benefits of vaccination, Ho et al. found that COVID-19 vaccination is less efficacious in MM patients, leading to increased rates of severe infection, hospitalization, ICU admission, and mortality even after vaccination [39]. While mortality rates, ICU admission, and hospitalization vary across studies, the increased risk remains consistent, as found by a 2024 meta-analysis, which also identified age and obesity as significant modifiers [36]. Advanced age, renal failure, active or progressing MM, lymphopenia, and comorbidities such as cardiovascular disease, diabetes, and chronic kidney disease were also found to independently predict worse outcomes [15,17,23,39].

Outcomes also vary based on treatment exposure and intensity and the response to therapy. Individuals with MM show significantly lower anti-spike antibody response rates, neutralizing antibody response rates, and T-cell responses after vaccination compared to the general population [18]. This combination of challenges in the treatment of COVID-19 in individuals with MM necessitates novel interventions that can safely mitigate severe viral infections without compromising MM outcomes and status.

Multiple therapeutic advances have modified the clinical management of COVID-19 over time, including the introduction of vaccination strategies, antiviral agents, and immunomodulatory therapies. However, clinically significant disease continues to occur, particularly in high-risk groups with impaired immune function. Patients with MM remain among those at persistently elevated risk due to disease-related and treatment-related immune dysfunction. Consequently, EV-based interventions should be considered as adjunctive strategies for persistently vulnerable immunocompromised subgroups, rather than replacements for established COVID-19 therapies.

In the modern MM treatment landscape, several therapeutic classes contribute to clinically relevant immune modulation. These include anti-CD38 monoclonal antibodies, BCMA-directed cellular therapies, antibody–drug conjugates, and bispecific T-cell engagers. Collectively, these treatments may impact humoral immunity, cellular immune surveillance, and vaccine responsiveness, thereby contributing to heterogeneity in infection risk and clinical outcomes.

### 3.2. Interleukin-6: A Shared Amplifier Within a Multidirectional Network

IL-6 activates downstream JAK1/JAK2-STAT, PI3K/AKT, and MAPK/ERK cascades through the membrane-bound receptor complex (IL-6Rα/gp130) [40]. Under normal physiological conditions, IL-6 coordinates acute-phase responses, hematopoiesis, and adaptive immune activation. In abnormal states, IL-6 drives a spectrum of diseases, particularly those operating through hyperinflammation such as severe malignancy or sepsis [41].

In MM, IL-6 is one of the driving factors for myeloma cell proliferation, inhibiting apoptosis and promoting transcription of anti-apoptotic proteins including MCL-1, BCL-2, and BCL-XL [42]. Beyond its anti-apoptotic and tumor-cell effects, IL-6 fundamentally remodels bone marrow by promoting angiogenesis via VEGF induction, activating osteoclasts to produce the lytic bone lesions characteristic of MM, and supporting the immunosuppressive cells that facilitate immune evasion [41,43]. IL-6 also drives hepatic production of acute-phase reactants (C-reactive protein, serum amyloid A, fibrinogen), contributing to a systemic inflammatory and hypercoagulable state characteristic of MM [40].

In the context of COVID-19 infection, IL-6 functions as one of the main drivers of the immune response. Viral infection triggers rapid IL-6 production and release from activated macrophages, dendritic cells, and pulmonary epithelial cells [44]. In one observational study, an elevated serum IL-6 threshold of >30 pg/mL was the strongest independent predictor of invasive mechanical ventilation (OR 7.1, *p* < 0.001), and untreated patients with elevated IL-6 had a hazard ratio for mortality of 4.6 (*p* = 0.003) [45].

IL-6-driven hyperinflammation in COVID-19 includes endothelial injury, complement system dysregulation, and the release of secondary mediators (IL-1β, TNF-α, IL-8). These components amplify and sustain the cytokine cascade, resulting in widespread microvascular thrombosis and angiogenesis in response to endothelial damage [46,47].

The concurrent occurrence of MM and COVID-19 creates a state of exaggerated IL-6 elevation and consequent inflammatory injury. The baseline increase in IL-6 present in MM is compounded by the inflammatory response to COVID-19 driven by macrophages and dendritic cells [34]. This exaggerated response forms part of the rationale for therapeutic strategies targeting IL-6 signaling in MM patients with COVID-19.

Despite the established importance of IL-6 in both MM and COVID-19, IL-6 operates within a multidirectional inflammatory environment rather than as a singular regulator of this pathway. Importantly, IL-6 blockade has not universally improved SARS-CoV-2 outcomes. The randomized evidence is explicitly heterogeneous: the double-blind, placebo-controlled COVACTA trial found no 28-day mortality benefit from tocilizumab, whereas the open-label RECOVERY and REMAP-CAP platform trials demonstrated a survival benefit confined to specific strata, hypoxic patients with C-reactive protein ≥75 mg/L in RECOVERY, and critically ill patients receiving organ support in REMAP-CAP [48,49,50]. This pattern indicates that inflammatory pathways operate in parallel rather than strictly downstream of IL-6. Other mechanisms of interaction include T-cell exhaustion, checkpoint ligand regulation, NK-cell functional impairment, complement dysregulation, endothelial injury, and macrophage polarization toward the M2 phenotype. It follows that for effective SARS-CoV-2 treatment in MM patients, strategies focusing solely on IL-6-axis suppression will be inadequate, and the observation that motivates the multi-node interventions is considered in Section 4, Section 5 and Section 6.

### 3.3. Immune Dysregulation and Viral Susceptibility

The bone marrow microenvironment constitutes the primary site of immune dysfunction in MM, functioning as an immune-remodeling niche that contributes to both disease progression and viral susceptibility. Myeloid-derived suppressor cells (MDSCs) accumulate within the bone marrow microenvironment and actively suppress T-cell and NK-cell effector function through arginase-1-mediated arginine depletion, reactive oxygen species generation, and PD-L1 upregulation, mechanisms that converge with the checkpoint exhaustion observed systemically in MM [51].

Resident marrow macrophages are reprogrammed toward an M2-like phenotype by tumor-derived IL-10, TGF-β, and direct myeloma-cell contact, impairing type I interferon (IFN-α/β) production and creating a permissive environment for SARS-CoV-2 replication. The bone marrow immune landscape is also spatially heterogeneous: intramedullary and extramedullary compartments harbor distinct microenvironments, with extramedullary disease associated with greater MDSC enrichment, regulatory T-cell accumulation, and immune escape. Critically, these defects are not restricted to active MM. MDSC accumulation, macrophage polarization shifts, and checkpoint-ligand upregulation are detectable even at the MGUS stage, [52] indicating that immune vulnerability precedes clinical disease onset and represents a progressively exploitable therapeutic target.

This early, microenvironment-anchored vulnerability is mechanistically continuous with the way MM evades therapy. Drug resistance in MM is not a static cell-intrinsic property but a dynamic stromal-adaptive process, encompassing soluble-factor-mediated drug resistance (SFM-DR) driven by IL-6, IGF-1, and related cytokines, and cell-adhesion-mediated drug resistance (CAM-DR) arising from integrin-mediated contact with bone marrow stromal cells, that is detectable from the MGUS stage and becomes progressively exploitable as disease advances [52]. The same niche that sustains these resistance programs also sustains the immune escape described above, which is why interventions aimed at the microenvironment, rather than at the plasma cell alone, are conceptually attractive in this population.

Beyond IL-6-mediated inflammation, MM patients demonstrate a profound humoral immunodeficiency that enhances susceptibility to severe viral infections. MM individuals experience suppression of normal B-cell maturation and polyclonal immunoglobulin synthesis, which is exacerbated by disease progression [53]. Anti-myeloma therapies compound this further, with anti-CD38 agents (daratumumab, isatuximab) depleting normal B-cell precursors and plasmacytoid dendritic cells along with the intended malignant plasma cells [54,55].

NK-cell function is reduced in MM, with diminished peripheral cell counts, cytotoxic activity, and IFN-γ production [56]. Daratumumab further contributes to reduced NK-cell function, with treated patients showing markedly reduced CD38+ NK cells in peripheral blood and bone marrow [55]. T-cell immunity exhibits increased expression of inhibitory checkpoints (PD-1, CTLA-4, BTLA), with PD-1 elevated across all disease phases and correlating with adverse outcomes on multivariate analysis. Treg expansion and declining Th1/Treg ratios reflect progressive T-cell erosion with advancing disease [54]. Dendritic cells follow a similar pattern, decreasing with MM progression. This leads to reduced antigen-presenting capacity and decreased production of the type I interferons necessary for early antiviral defense against respiratory coronaviruses [57].

The culmination of these immune dysfunctions creates opportunity for viral replication, prolonged viral shedding, and increased risk of new variants. MM patients demonstrate significantly prolonged COVID-19 RNA detectability, B-cell and T-cell depletion, and viral shedding compared to immunocompetent hosts [58]. This prolonged replication creates conditions for increased viral evolution. Immunosuppressed patients demonstrate an increased rate of novel single nucleotide polymorphisms (SNPs) [59,60]. This raises concerns about individual outcomes of viral infection as well as the risk of immunocompromised hosts becoming possible sources of new viral variants.

### 3.4. Synthesis and Therapeutic Implications

The convergence of mechanisms described in this section suggests a pathophysiological pathway that is resistant to single-target interventions. While IL-6 constitutes a critical part of the disease process, it operates within a broader network including endothelial injury, immune-cell dysfunction, and coagulatory effects. Mapped onto the tier framework introduced above, IL-6 spans tiers (i) and (ii), but the defects of tier (iii) and several parallel mediators of tier (ii) lie outside its reach, which is the structural reason single-cytokine blockade has underperformed.

This complexity must be taken into account when devising new therapies. Interventions that target a single cell type, pathway, or cytokine may leave gaps in the treatment of such a multidimensional disease. What is required is an intervention capable of addressing the majority of the cellular processes that occur in SARS-CoV-2 infection.

Therapeutic exposures further modify the immune environment in ways clinically relevant to SARS-CoV-2 susceptibility and vaccine failure. Anti-CD38 monoclonal antibodies such as daratumumab and isatuximab target malignant plasma cells but also affect various CD38-expressing immune populations, including NK cells, regulatory T cells, regulatory B cells, and subsets of activated T cells [55]. A key consequence is significant NK-cell depletion via CD38-mediated fratricide, which may impair early antiviral cytotoxic responses and diminish immune monitoring [55]. This results in a paradoxical immunologic state: while anti-CD38 therapy may reduce immunosuppressive regulatory compartments and enhance anti-myeloma immunity, it also depletes innate effector populations essential for rapid viral control. In the context of SARS-CoV-2 infection, these effects may delay viral clearance, prolong viral shedding, and diminish vaccine-induced cellular immunity.

BCMA-directed therapies further complicate immune disruption. Because BCMA is present on both normal and malignant plasma cells, BCMA-targeted CAR T-cell therapy, antibody-drug conjugates, and bispecific antibodies can cause profound plasma cell aplasia, hypogammaglobulinemia, and impaired humoral reconstitution. The magnitude of this effect is now quantifiable: in a retrospective cohort of patients receiving anti-BCMA bispecific antibodies, profound hypogammaglobulinemia occurred in 100% of responders and persisted throughout treatment, while immunoglobulin replacement was associated with a roughly ten-fold lower rate of grade 3–5 infection (incidence rate ratio 0.10; 95% CI 0.01–0.80; *p* = 0.0307) [12]. These effects are especially significant for respiratory viral infections, where neutralizing antibody responses are critical for protection against severe disease and reinfection. In patients undergoing BCMA-directed therapy, inadequate serologic responses to SARS-CoV-2 vaccination may result from both disease-related immune paresis and treatment-induced depletion of antibody-producing cells.

T-cell redirecting therapies, including BCMA- and GPRC5D-directed bispecific antibodies, are increasingly used in relapsed or refractory MM and warrant careful consideration in the context of infectious risk. These agents rely on activation of endogenous T cells for antitumor activity and therefore induce significant immune modulation. This may have implications for overall immune homeostasis, including antiviral responses. In the setting of COVID-19, where outcomes are influenced by both early viral control and subsequent inflammatory injury, such therapies may contribute to heterogeneous clinical risk profiles. As a result, patients receiving these agents may represent a subgroup that warrants dedicated evaluation in studies of infection outcomes and investigational immunomodulatory strategies such as EVs.

## 4. Mesenchymal Stem Cells and Extracellular Vesicles as Therapeutic Modalities

### 4.1. Mesenchymal Stem Cells: Cellular Immunomodulators

Mesenchymal stem cells (MSCs) are multipotent adult stromal cells present in almost all human tissues, possessing capacities for immunomodulation, reduction in inflammation, and tissue regeneration [61,62]. The immunomodulatory functions of MSCs have long been theorized to be driven by paracrine signals, which may in turn be a function of EVs [62]. This is clinically significant given the emerging identification of EVs not merely as functional units of MSC therapy, but as standalone therapeutic agents separate from their cellular source.

In the context of COVID-19, MSC therapy shows promise through suppression of pro-inflammatory cytokines (IL-6, IL-1β, TNF-α), enhancement of anti-inflammatory mediators, and promotion of tissue repair and regeneration [61,63]. Early clinical trials in moderate-to-severe COVID-19 have shown safety and preliminary signals of activity with respect to inflammation, oxygenation indices, and radiographic improvement [64].

However, there are limitations to the use of MSCs and their EVs in patients with MM. Pulmonary first-pass trapping reduces the efficacy of interventions and may cause microvascular obstruction, as the large size of MSCs leads to mechanical sequestration in the pulmonary capillary bed [65,66]. There are also challenges of proper storage, maintenance of viability, and standardization of therapy [67]. All of these issues reduce the bioavailability and efficacy of MSCs and position cell-free EV-based therapies as a potentially more bioavailable alternative.

### 4.2. Extracellular Vesicles: Cell-Free Therapeutic Platform

EVs are membrane-bound particles (30–1000 nm) released by practically all cell types and responsible for a variety of physiological functions [68]. EVs mediate intercellular communication through the transport of proteins, lipids, mRNA, miRNA, and other bioactive cargo, with function varying based on cell type and origin [69,70].

The rationale for utilizing EVs as therapeutic agents rests first on their capacity to preserve the immunomodulatory function of their parent MSCs, with the added benefit of reduced immunogenicity [71]. Owing to their small size, EVs cross biological barriers, including the alveolar–capillary interface, that are inaccessible to intact cells such as MSCs [70]. Because of their extracellular nature, EVs carry minimal MHC class I/II surface expression, significantly reducing immunogenic risk and bypassing the matching requirements associated with cellular therapies [72].

EVs are broadly categorized into exosomes, microvesicles, and apoptotic bodies based on their biogenesis and size. Exosomes are small, nano-sized vesicles (approximately 30–100 nm in diameter) that originate from the endosomal system. They form through inward budding of late endosomal membranes, leading to the formation of intraluminal vesicles within multivesicular bodies, which are subsequently released into the extracellular space following fusion with the plasma membrane [68]. MVs are larger vesicles, typically ranging from approximately 100–1000 nm, and are generated by direct outward budding and shedding of the plasma membrane. Apoptotic bodies are the largest EV subtype, formed during programmed cell death, and can contain cytoplasmic components and fragmented nuclear material. These vesicle populations differ in their biogenesis pathways, size distribution, and molecular cargo, which contributes to variability in their biological roles and functional effects across physiological and pathological contexts [68].

In line with MISEV2023, which discourages the term “exosome” unless endosomal biogenesis has been demonstrated, we use the operational term “small EVs (sEVs)” when biogenesis is not established; where trial sponsors designate a product as “exosomes,” that source terminology is retained verbatim for fidelity to the original report [73]. Critically, most clinical EV preparations, regardless of label, are heterogeneous mixtures of these subtypes, as current isolation methods cannot yield pure populations. This is not merely a labeling problem: the isolation method itself shapes the product, since differential ultracentrifugation, tangential-flow filtration, and size-exclusion chromatography yield different sEV-to-microvesicle ratios and different burdens of co-isolated protein and lipoprotein contaminants, which in turn alter the apparent biological effect attributed to a given preparation [74]. This compositional variance limits mechanistic interpretation across studies and complicates dose standardization, reproducibility, and regulatory evaluation (Table 1).

### 4.3. Mechanisms of EV-Mediated Immunomodulation

The therapeutic potential of EVs is mediated through anti-inflammatory action, immunomodulation, and cargo transfer of biologically active compounds, both to reduce the effects of COVID-19 and to promote tissue regeneration during recovery [72]. Through cytokine modulation and the transfer of immunoregulatory miRNAs and proteins, EVs help reduce inflammation while maintaining immune function [75].

MSC-derived EVs reprogram macrophages from a pro-inflammatory M1 phenotype to an anti-inflammatory M2 phenotype via miRNA cargo transfer, with miR-146a the best-supported mediator and a contributory role proposed, though less firmly established, for miR-145 [75,76]. EVs also modulate T-cell responses by inducing regulatory T cells (Tregs) through IL-10 signaling, suppressing Th17 differentiation, and inhibiting CD4+ and CD8+ T-cell activation [77].

Beyond immunomodulation, EVs promote vascular repair and tissue regeneration through the transfer of angiopoietin-1 mRNA and pro-angiogenic miRNAs, which restore endothelial permeability and stimulate endothelial proliferation [78].

**Figure 2:**
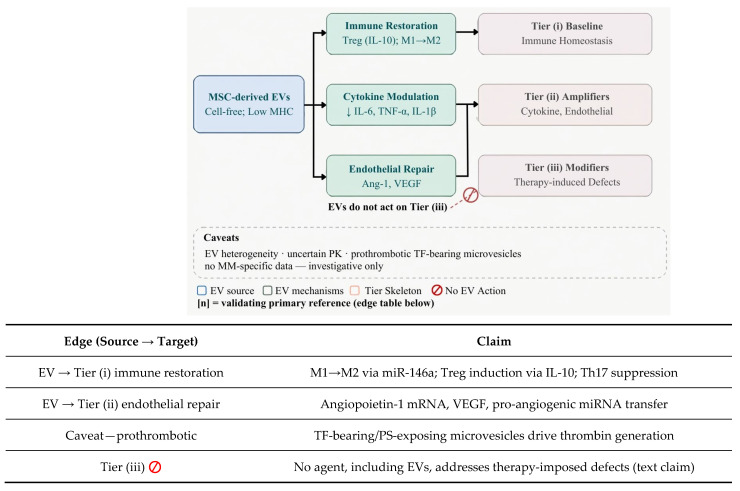
Mechanistic model illustrating the points at which mesenchymal stem cells (MSCs) and their extracellular vesicles (EVs) intersect the MM–SARS-CoV-2 network. This figure maps EV actions onto the tier framework of Figure 1, showing EV-mediated effects across Tier (i) (immune-homeostasis restoration) and Tier (ii) (cytokine modulation, endothelial repair, coagulation modulation), while indicating that Tier (iii) therapy-imposed defects are not addressed by EVs.

With the mechanistic role of EV-mediated immunomodulation and the structural advantages of cell-free delivery over intact MSCs established, the question remains whether early clinical evidence supports translating this theoretical mechanism into practice, specifically for high-risk immunocompromised populations such as those with MM (Figure 2).

## 5. Clinical Evidence for EV Therapy in Severe COVID-19

### 5.1. Rationale for EV Therapy in COVID-19

The prognosis of COVID-19 depends on multiple immune mechanisms. In early infection, mechanical viral clearance and cytokine-mediated responses form the mainstay of immunological defense; failure of these initial protective mechanisms allows progression to severe COVID-19 [79]. Severe COVID-19 is characterized by a simultaneous increase in multiple inflammatory cytokines, including interleukins (IL-1, IL-6, and others), TNF-α, interferons (IFN-β, IFN-λ), MCP-1, and MIP-1α [79]. This response is not without consequence; the cytokine storm and associated host inflammatory responses lead to endothelial injury, acute respiratory distress syndrome, and reduced lung function [80]. With different phases of COVID-19 operating through distinct pathologic mechanisms, therapeutic targets are difficult to pinpoint, and the timing of antivirals and immunomodulatory therapies affects both efficacy and potential toxicity [80,81]. The difficulty of therapy is further augmented by the multi-organ effects of COVID-19, making single-organ therapeutic targets impractical [82].

In MM, beyond the usual challenges of COVID-19 therapy, the immunodeficiency caused by both the plasma cell dyscrasia and its treatment adds complexity and leads to higher rates of hospitalization, severe COVID-19, and mortality compared to immunocompetent patients [17].

Current COVID-19 management has improved considerably with vaccination, early antiviral therapy, corticosteroids, IL-6 inhibition, JAK inhibition, anticoagulation strategies, and optimized supportive care. However, these interventions do not completely resolve the persistent challenges of impaired viral clearance, treatment-related immune dysfunction, endothelial injury, coagulopathy, and tissue-repair failure in high-risk MM patients [83]. Current regimens largely repurpose immunomodulating agents developed for immunocompetent patients (dexamethasone, protease inhibitors, IL-6 and JAK inhibitors), which do not address all mechanisms of injury in COVID-19 and which the European Myeloma Network consensus identifies as requiring myeloma-specific adaptation [84]. While the IL-6 inhibitor tocilizumab and the JAK1/2 inhibitor baricitinib have shown promise in case reports of cancer patients with COVID-19, they require further study, as positive outcomes coexist with reports of toxicity, increased infection risk, and excess immunosuppression [85,86,87].

**Figure 3:**
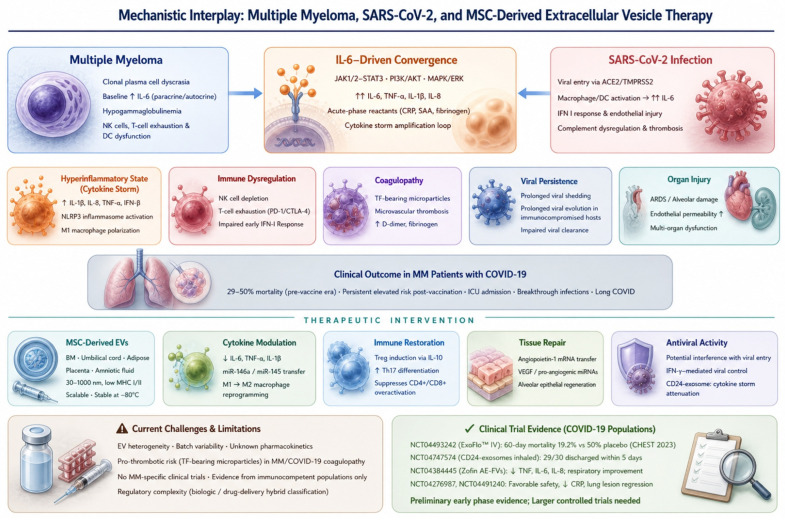
Mechanistic interplay between multiple myeloma (MM), SARS-CoV-2 infection, and the proposed therapeutic actions of mesenchymal stem cell (MSC)-derived extracellular vesicles (EVs). MM establishes a baseline state of immune dysfunction and IL-6–primed inflammation that is further amplified by SARS-CoV-2 infection through cytokine production, complement activation, endothelial injury, and coagulation abnormalities, resulting in cytokine storm, impaired antiviral immunity, viral persistence, organ injury, and adverse clinical outcomes. MSC-derived EVs are proposed to mitigate these processes through multimodal actions, including suppression of pro-inflammatory cytokines, restoration of immune homeostasis, promotion of tissue repair, and potential antiviral effects. Although biologically plausible, these therapeutic mechanisms remain investigational, as current evidence is derived from non-MM COVID-19 populations and no MM-specific clinical trials have been conducted. Created using Illustrae (https:/Illustrae.co/ accessed on 14 June 2026) an AI-powered illustration tool.

EVs occupy an advantageous position in this context. They are capable of multi-pathway effects, carrying miRNAs, proteins, and lipids to simultaneously reduce hyperinflammation, promote endothelial repair, and modulate coagulation [88]. In the early phase of COVID-19, EVs may inhibit viral replication and regulate the cytokine storm; in later phases, they may promote alveolar repair, angiogenesis, and resolution of fibrosis [88,89]. These attributes make EVs particularly relevant to COVID-19 treatment in MM patients, where disease severity is exacerbated by B-cell and T-cell immunodeficiency, treatment-induced immunosuppression, and inflammation [90] (Figure 3).

### 5.2. Clinical Trial Evidence

No clinical trial has enrolled MM patients to study EVs in COVID-19; the evidence base derives entirely from non-MM, predominantly immunocompetent populations, and must be read with that limitation in mind [63,91,92]. The most informative randomized dataset is the phase 2 ExoFlo trial (NCT04493242) of intravenous bone marrow MSC-derived EVs in COVID-19-associated moderate-to-severe ARDS. Its pre-specified primary endpoint, all-cause 60-day mortality in the intention-to-treat population, was not met (χ^2^, *p* = 0.1343). The frequently cited reduction in 60-day mortality from 50% to 19.2% derives from an exploratory post hoc subgroup restricted to patients aged 18–65 years with respiratory failure (relative risk 0.385; 95% CI 0.159–0.931; *p* = 0.0340), a finding the accompanying CHEST editorial cautioned should be interpreted with great caution [93]. The flagship randomized EV trial therefore illustrates why the current evidence is hypothesis-generating rather than confirmatory.

Amniotic fluid–derived EVs, delivered intravenously, have shown preliminary, uncontrolled signals of activity in preventing progression of mild-to-moderate COVID-19, with acceptable short-term safety [94]. Pilot studies of nebulized EVs derived from umbilical cord MSCs, and of placental-derived EVs in critically ill COVID-19, have likewise reported early signals of activity that remain uncontrolled and require confirmation [63,91,95].

The available evidence base is also subject to important structural limitations that must be acknowledged alongside the reported findings. Publication bias is a substantial concern: small, single-center studies with positive or neutral safety signals are disproportionately represented in the published record compared to inconclusive or negative results. Several trials listed in Table 2 remain ongoing or have not reported primary efficacy endpoints, precluding comprehensive assessment of treatment effect. The absence of reported failures in the published EV literature should not be interpreted as evidence of consistent benefit; rather, the current evidence base should be understood as preliminary, hypothesis-generating, and subject to the distortions inherent to early-phase trial reporting. Pre-registration of future EV trials and strict adherence to CONSORT reporting standards are necessary mitigations.

### 5.3. Safety Profile and Limitations of EV-Based Therapy

Preliminary clinical studies of MSC-derived EVs in COVID-19 have indicated favorable short-term safety; however, the current evidence base is limited and should be interpreted with caution [93]. Most studies are small, heterogeneous in design, and conducted in non-MM or predominantly immunocompetent COVID-19 populations, which restricts direct extrapolation to patients with plasma cell dyscrasias, treatment-related lymphopenia, hypogammaglobulinemia, or recent exposure to anti-CD38 or BCMA-directed therapies. Consequently, while EVs may present theoretical advantages over parent MSCs, their safety and success in MM-specific clinical contexts remain unestablished.

A major limitation of EV-based therapy is product heterogeneity. EV preparations can vary depending on the parent cell source, donor-related differences, culture conditions, and the methods used for isolation and storage. This variability contributes to differences in EV composition, including proteins, lipids, nucleic acids, and surface molecules, which in turn may influence their biological activity. Such inconsistencies make it difficult to directly compare results across studies and pose challenges for standardization and reproducibility. In addition, the lack of universally accepted classification and standardized manufacturing and quality control frameworks continues to limit clinical translation of EV-based therapeutics [74].

Pharmacokinetic and biodistribution profiles of EVs are not yet fully characterized. EVs are often proposed to have favorable tissue distribution compared with parent cells such as mesenchymal stem cells, but the extent and consistency of their in vivo localization following systemic administration are not fully defined. Their distribution is likely influenced by multiple factors, including route of administration, particle size, surface molecular composition, and host physiological or inflammatory status, as well as clearance by the mononuclear phagocyte system. As a result, key parameters such as optimal dosing, timing of administration in relation to disease stage, treatment frequency, and targeting efficiency remain insufficiently standardized and require further systematic evaluation.

Potential pro-thrombotic effects of EVs warrant careful evaluation, particularly in patients with MM and severe COVID-19, both of which are independently linked to hypercoagulability. The mechanism is specific rather than generic: tissue-factor–bearing microvesicles can trigger the extrinsic coagulation cascade by complexing with factor VIIa, while phosphatidylserine-exposing vesicle surfaces provide a platform for prothrombinase (factor Xa–Va) assembly and accelerated thrombin generation [96]. Although MSC-derived EVs may also exert anti-inflammatory and endothelial-stabilizing effects, the biological heterogeneity of EV preparations means their net thrombotic impact may vary with source, purification method, cargo, and patient context. This matters acutely in MM–COVID-19 co-occurrence, which represents a convergence of three independent prothrombotic pressures, MM-associated hypercoagulability, COVID-19-associated coagulopathy, and the thrombotic risk of immunomodulatory drugs such as lenalidomide and pomalidomide, so that even a modest procoagulant contribution from an EV product could be clinically consequential in precisely the population proposed for treatment. Future trials should therefore systematically monitor coagulation markers, thrombotic complications, and interactions with anticoagulation strategies.

### 5.4. Application to MM Population

The minimal immunogenicity, low toxicity, and multidirectional mechanism of EVs position them as theoretically suitable candidates for investigation in immunocompromised MM patients [90]. Concurrent anti-MM therapy may also be feasible, with EVs potentially forming part of the anti-cancer regimen given their capacity to promote an anti-inflammatory state [97]. Furthermore, the cell-free nature of EVs makes them scalable and accessible, and they can be genetically engineered to carry specific compounds or target specific cells (endothelial cells, alveolar type II epithelial cells, and alveolar macrophages), allowing localized immunomodulation and tissue repair at sites of injury [98].

It must be explicitly stated, however, that these theoretical advantages remain unvalidated in MM-specific clinical settings. No published trial has enrolled MM patients for EV-based therapy in the context of COVID-19 or any respiratory viral infection. The properties described above represent mechanistically plausible advantages, not established clinical benefits. EVs in this context should be framed as an investigational strategy warranting rigorous disease-specific evaluation, not a treatment ready for clinical integration.

## 6. Future Directions and Research Priorities

### 6.1. Mechanistic Studies

Despite encouraging early-phase data, much remains to be understood regarding the mechanism of action of EVs specifically in MM. Studies are needed to clarify the particles and their therapeutic targets, enabling more targeted treatment and improved efficacy.

Identifying which cargo components (miRNA, mRNA, proteins) are responsible for the immunomodulatory effects can aid in engineering more targeted treatments. In particular, it should be explored whether EVs can at least partially restore the innate antiviral function lost through daratumumab-induced NK-cell fratricide [55]. Furthermore, directing EVs toward specific cellular targets may help determine which EV designs best alleviate the disease burden of COVID-19 [90,99].

Future EV workflows should adhere to the MISEV (Minimum Information for Studies of Extracellular Vesicles) and MIQE (Minimum Information for Publication of Quantitative Real-Time PCR Experiments) guidelines to improve reproducibility and standardization [100]. While recent studies have followed MISEV and MIQE, adherence has been variable and reporting inconsistent [73,101].

Future studies should also explore combinations of established and emerging COVID-19 treatment protocols with EVs to identify optimal routes of therapy. Much remains unexplored regarding the timing of EV administration relative to MM treatment cycles. COVID-19 disease course is often described in phases with differing dominant pathophysiology, which may have implications for timing of EV-based interventions. In earlier stages characterized by active viral replication, interventions that modulate innate antiviral responses may be more relevant, whereas later stages marked by systemic inflammation and endothelial injury may require approaches aimed at immune regulation and tissue repair. However, the translational relevance of EV cargo in specifically targeting these phases remains theoretical and requires further validation.

In MM, this temporal framework is further complicated by treatment-related immune dysfunction. Periods following therapies such as anti-CD38 antibodies, CAR T-cell therapy, or bispecific T-cell engagers are associated with variable degrees of lymphocyte depletion and impaired humoral and cellular immunity. These intervals may therefore represent phases of increased susceptibility to viral infections, although the precise duration and clinical impact vary depending on treatment regimen and patient-specific immune recovery. Future EV trials in MM should therefore incorporate biomarker-guided dosing using serial IL-6, CRP, lymphocyte-subset recovery, and neutralizing antibody titers to align intervention timing with the patient’s immune state rather than fixed empirical schedules.

### 6.2. Clinical Trial Priorities

For EVs to be integrated into routine clinical practice for MM patients with COVID-19, trials designed specifically for MM patients are required. While initial studies have shown safety and preliminary activity in general COVID-19 populations, none have focused solely on MM patients [91,93]. Future trials should stratify patients by treatment exposure, including proteasome inhibitors, immunomodulatory drugs, anti-CD38 antibodies, BCMA-directed CAR T-cell therapy, antibody-drug conjugates, and bispecific or other T-cell redirecting antibodies. These therapies induce specific immune defects, NK-cell depletion, plasma cell aplasia, hypogammaglobulinemia, T-cell dysfunction, and impaired vaccine responsiveness, that may affect both COVID-19 susceptibility and response to EV-based therapy. Newly diagnosed patients may warrant separate study from those who have undergone multiple cycles of therapy.

These measures are needed from a public-health standpoint, as shown by EPICOVIDEHA and other cohort data demonstrating persistently elevated mortality through consecutive viral waves, even after widespread vaccination [14,16]. With MM patients also at elevated risk for long COVID and persistent post-COVID symptoms, there is a need to include them in clinical trials exploring the post-acute sequelae of COVID-19 [84]. Trials should also prioritize identification of validated predictive and pharmacodynamic biomarkers to guide patient selection and monitor treatment response [73].

### 6.3. Manufacturing and Regulatory Considerations

Successful clinical translation of EV-based therapeutics will require rigorous standardization of manufacturing, characterization, storage, and potency testing. Current preparations differ by parent-cell source, donor variability, culture medium, isolation platform, purification strategy, and cryopreservation conditions, all of which can influence cargo composition and biological activity. Adoption of MISEV and MIQE reporting standards is essential to increase reproducibility, minimize inter-study variability, and support regulatory evaluation [73,101]. Reporting guidelines alone are insufficient, however; future studies should also implement validated potency assays, quantitative release criteria, sterility testing, particle characterization, cargo profiling, and stability assessments under various storage conditions [102,103].

Regulatory development is further complicated by the intermediate classification of EVs, which share characteristics with biologic products, drug-delivery platforms, and cell-derived therapeutics. Unlike conventional small molecules, EVs carry complex, dynamic molecular cargo, making it difficult to identify a single active ingredient or mechanism of action. This regulatory ambiguity is itself documented in the manufacturing and policy literature, [74] and is especially relevant in MM, where therapeutic efficacy may depend on concurrent anti-inflammatory, antiviral, endothelial-stabilizing, and tissue-repair effects. Accordingly, future MM-focused trials should predefine clinically relevant biomarkers of activity, IL-6, CRP, ferritin, D-dimer, lymphocyte subsets, neutralizing antibody responses, and markers of endothelial injury, and assess product-specific pharmacokinetics and biodistribution.

### 6.4. Broader Applications in Immunocompromised Populations

The mechanisms by which EVs act suggest broader potential across immunocompromised populations [104,105]. Patients with other hematological malignancies, recipients of allogeneic hematopoietic cell transplantation, and individuals on B-cell-depleting or immunosuppressive therapies for autoimmune disease share similar vulnerabilities in immune response and disproportionately severe outcomes from respiratory viral infections such as influenza, respiratory syncytial virus, and adenovirus [105].

Establishing MM as the index population for this line of investigation could offer insight into translating EV therapy into a broader range of immunocompromised disease states.

## 7. Conclusions

The convergence of MM and COVID-19 represents a uniquely high-risk clinical intersection defined by profound immune dysregulation and an amplified inflammatory response. A prominent shared amplifier of this interaction is the IL-6 axis, which intensifies systemic inflammation and contributes to adverse outcomes; as developed throughout this review, however, IL-6 acts within a redundant, multidirectional network rather than as the single governing driver. Despite advances in vaccination and supportive care, patients with MM continue to experience suboptimal immune protection and remain highly vulnerable to severe SARS-CoV-2 infection and its complications.

Emerging evidence supports the theoretical potential of MSC-derived EVs as a novel, cell-free strategy that may simultaneously target multiple pathogenic pathways. By modulating cytokine responses, restoring immune homeostasis, and promoting tissue repair, EVs offer conceptual advantages over conventional single-target immunomodulatory therapies. Their favorable short-term safety profile, low immunogenicity, and potential compatibility with ongoing anti-myeloma treatment further support their investigation in this complex population.

However, translation into routine practice requires rigorous mechanistic studies, standardized manufacturing protocols, and well-designed clinical trials specifically targeting MM patients. Ultimately, EV-based therapies may eventually contribute to the management of severe viral infections in immunocompromised hosts, potentially extending beyond COVID-19 to broader infectious and inflammatory conditions.

The mechanistic properties of MSC-derived EVs, multimodal cytokine modulation, macrophage reprogramming toward an anti-inflammatory M2 phenotype, restoration of endothelial integrity, and promotion of alveolar repair, align conceptually with the multi-node pathophysiology described in this review. Unlike single-target agents such as IL-6 or JAK inhibitors, EVs theoretically address several pathogenic processes simultaneously, which is precisely the property required in a condition as immunologically complex as MM co-infected with SARS-CoV-2. This mechanistic alignment, however, must not be conflated with clinical efficacy. The existing evidence derives entirely from early-phase studies in non-MM, predominantly immunocompetent populations, is subject to publication bias, includes a flagship randomized trial whose pre-specified primary endpoint was not met, and cannot be directly extrapolated to the MM-specific immune landscape. EVs should therefore be positioned as a mechanistically compelling but investigational strategy. While well-motivated by existing pathophysiology, this approach demands rigorous, MM-specific clinical evaluation before any adoption into practice.

## Figures and Tables

**Table 1:** Comparative features of mesenchymal stem cells and extracellular vesicles.

Feature	Mesenchymal Stem Cells (MSCs)	Extracellular Vesicles (EVs)	Quantitative Comparator
Nature	Live cellular entity	Cell-free, biologically derived membrane nanovesicles	Not applicable
Source	Isolated from tissues (bone marrow, umbilical cord, adipose tissue, placenta)	Secreted by MSCs and other cell types	Not applicable
Size	10–30 µm	30–1000 nm (small EVs: 30–150 nm)	~100–1000× smaller
Primary therapeutic mechanism	Paracrine signaling (primarily via EV secretion)	Direct cargo delivery (proteins, miRNAs, lipids, mRNAs)	Not established
Immunogenicity	Moderate; allogeneic use generally tolerated but may elicit responses with repeated dosing	Low; minimal MHC expression, reduced immunogenic potential	Not established
Safety considerations	Potential lung entrapment/embolism due to cell size; risk of senescence or transformation in long-term culture	Minimal embolic risk; no replicative potential; no malignant-transformation capacity	Not established
Manufacturing & storage	Requires cryopreservation; viability concerns; complex culture/expansion	Stable at −80 °C or lyophilized; amenable to large-scale manufacturing	Not established
Batch-to-batch consistency	Significant variability with donor, passage, culture conditions	More consistent under standardized conditions; amenable to QC testing	Not established
Biological barriers	Limited capacity to cross barriers (blood–brain barrier, placenta)	Enhanced barrier crossing due to nanoscale size and surface properties	Not established
Pharmacokinetics/half-life	Pulmonary first-pass trapping; rapid sequestration	Biodistribution and circulating half-life poorly characterized in humans	Not established
Dose–response	Variable; cell-number based	No validated dose metric (particle number vs. protein mass)	Not established
Drug interactions	Limited data; potential interactions with immunosuppressants	Minimal documented drug–drug interactions to date	Not established
Regulatory status	Several approved products for specific indications (e.g., graft-versus-host disease)	No regulatory approvals; multiple products in clinical development	Not applicable

**Table 2:** Representative clinical trials of extracellular vesicles in COVID-19.

NCT Number	EV Type	Phase	Population	Route	Key Outcomes/ Findings	Limitations/Level of Evidence
NCT04276987	Adipose MSC-derived EVs	I	Severe COVID-19 pneumonia	Aerosol inhalation	2 × 10^8^ nanovesicles/3 mL × 5 days; well tolerated, no pre-specified AEs; modest lymphocyte increases; CT lesion regression	Uncontrolled phase I; primary endpoint safety/tolerability; efficacy not assessable
NCT04313647	Adipose MSC-derived EVs	I	Healthy volunteers	Aerosol inhalation	Dose escalation up to 16 × 10^8^ particles; two non-serious AEs at 7 days; establishes inhaled tolerability ceiling	Healthy volunteers; no efficacy endpoint; not a patient population
NCT04491240	MSC-derived sEVs (“exosomes” per sponsor)	I/II	COVID-19, PCR-confirmed (18–65 y)	Inhalation	0.5–2 × 10^10^ particles BID × 10 days; no moderate/severe AEs within 30 days; improved CRP; efficacy endpoints unchanged vs. placebo	Efficacy endpoints NOT met vs. placebo; small; controlled
NCT04493242	ExoFlo™ (bone marrow MSC EVs)	II	Moderate-to-severe COVID-19 ARDS	Intravenous	Pre-specified ITT primary endpoint (60-day mortality) NOT met (*p* = 0.1343); 50% vs. 19.2% reduction limited to post hoc subgroup aged 18–65 with respiratory failure (RR 0.385, 95% CI 0.159–0.931, *p* = 0.0340)	PRIMARY ENDPOINT NOT MET; mortality benefit post hoc subgroup only; CHEST editorial urged caution; controlled phase II
NCT04384445	Zofin (human amniotic fluid EVs)	I/II	Moderate-to-severe COVID-19 ARDS	Intravenous	2–5 × 10^11^ particles/mL on days 0, 4, 8; reported safe; suppression of cytokines (TNF, IL-6, IL-8) and respiratory improvement in case series	Case-series/early-phase signal; uncontrolled; no formal efficacy endpoint met
NCT04389385	COVID-19-specific T-cell-derived EVs	I	Early stage COVID-19	Inhalation	T cells activated with SARS-CoV-2 peptides in vitro; 2 × 10^8^ exosomes 5× daily × 5 days; IFN-γ-mediated viral control proposed; results pending	Phase I; results not reported; efficacy not assessable
NCT04747574	CD24-exosomes, engineered HEK-293 (Ichilov, Israel)	II	Moderate-to-severe COVID-19/cytokine-storm risk	Inhalation (once daily × 5 days)	Open-label dose escalation; phase I: 29/30 discharged within 5 days; targets cytokine release syndrome	Open-label, uncontrolled; non-randomized; primary efficacy not yet established
NCT04969172	CD24-exosomes (Athens Medical Society, Greece)	II	Moderate-to-severe COVID-19	Inhalation (once daily × 5 days)	Randomized, double-blind, placebo-controlled; parallel arm to NCT04747574; primary outcome: cytokine-storm attenuation	Controlled; outcome data not yet reported
NCT05354141	ExoFlo™, bone marrow MSC EVs (Direct Biologics)	III	Moderate-to-severe ARDS (any etiology incl. COVID-19)	Intravenous	Three doses ExoFlo 15 mL vs. placebo; primary endpoint 60-day all-cause mortality; secondary VFDs, oxygen-free days, ICU-free days; ongoing	Ongoing; primary endpoint not yet reported; controlled phase III

The ExoFlo entry (NCT04493242) records that the pre-specified intention-to-treat primary endpoint was not met and that the reported mortality reduction is a post hoc subgroup finding.

## Data Availability

No patient data were directly utilized in this study.

## Abbreviations

The following abbreviations are used in this manuscript:

AE	Adverse event
ARDS	Acute respiratory distress syndrome
BCMA	B-cell maturation antigen
BID	Twice daily
CAR	Chimeric antigen receptor
CI	Confidence interval
COVID-19	Coronavirus disease 2019
CRP	C-reactive protein
EV	Extracellular vesicle
GPRC5D	G protein-coupled receptor family C group 5 member D
IFN	Interferon
IL	Interleukin
ITT	Intention-to-treat
JAK	Janus kinase
MHC	Major histocompatibility complex
miRNA	MicroRNA
MM	Multiple myeloma
MSC	Mesenchymal stem cell
NK	Natural killer
PCR	Polymerase chain reaction
RR	Relative risk
SARS-CoV-2	Severe acute respiratory syndrome coronavirus 2
sEV	Small extracellular vesicle
TGF-β	Transforming growth factor-beta
TNF	Tumor necrosis factor
VFD	Ventilator-free days
